# Follow‐Up Magnetic Resonance Imaging in Monitoring Charcot Foot and Its Association With Total Contact Cast Treatment Duration and Long‐Term Outcomes: A Retrospective Cohort Study

**DOI:** 10.1002/jfa2.70058

**Published:** 2025-06-21

**Authors:** Johan Schoug, Per Katzman, Erik Uddman, Magnus Löndahl

**Affiliations:** ^1^ Department of Endocrinology Skåne University Hospital Lund Sweden; ^2^ The Institution for Clinical Sciences in Lund Faculty of Medicine Lund University Lund Sweden

**Keywords:** Charcot arthropathy, cohort, diabetic foot, magnetic resonance imaging, treatment

## Abstract

**Aims/Hypothesis:**

Charcot foot (CF), a potentially debilitating complication of neuropathy, requires offloading to avoid foot deformities. Follow‐up MRI examinations are often used to determine the optimal duration of total contact cast (TCC) offloading treatment. This study investigated the use of follow‐up MRI during CF treatment and its relationship to offloading duration and risk of future surgery.

**Methods:**

People with diabetes mellitus and MRI‐confirmed CF treated at Skåne University Hospital (Lund and Malmö, Sweden) between 2006 and 2022 were studied retrospectively. Individuals monitored with follow‐up MRI examinations were compared with those who only underwent diagnostic MRI. A regression model was applied to evaluate factors predicting TCC and total CF treatment duration.

**Results:**

One‐hundred and twenty‐two individuals (45 [37%] DM1; 47 [39%] women; median age 60 [IQR 53–68] years) with a total of 143 CF events were included. 76 (53%) of these CF events were monitored using a total of 141 follow‐up MRI examinations. Individuals monitored with MRI had significantly longer TCC and total CF treatment durations (*p* < 0.001). Individual characteristics (with the exception of sex), rate of stage 1 CF, and risk of future surgery did not differ between the two groups and only use of follow‐up MRI (*p* < 0.001) remained a significant predictor of both longer TCC and total CF treatment durations in a regression model.

**Conclusions/Interpretation:**

In this retrospective cohort study, use of follow‐up MRI was associated with longer TCC and total treatment times despite similar characteristics and outcomes. Prospective studies are needed to further elucidate the optimal use of MRI in monitoring CF.

## 1 Introduction

Charcot's neuropathic osteoarthropathy or Charcot foot (CF) is an uncommon and potentially debilitating complication of neuropathy, nowadays most often preceded by diabetes mellitus [1]. Diagnosing and treating CF in its earliest stage is associated with improved outcomes [2, 3, 4, 5]. However, in its earliest stage, differential diagnostics are challenging as X‐ray examinations are negative, and radiological confirmation requires magnetic resonance imaging (MRI) or other special imaging techniques [6]. Once confirmed, treatment of active CF with an offloading device, typically a total contact cast (TCC) continues until waned clinical signs of inflammation with normalized skin temperature as assessed on regular follow‐up visits [7, 8].

Studying and defining remission of active CF have received less scientific attention, and the use of follow‐up MRI examinations has been suggested to support decision‐making [9]. However, MRI is expensive, and its availability may be limited. Recently published guidelines concluded that there is a paucity of studies regarding the optimal use of radiological examinations in monitoring CF [7], but a few studies have shown potential usefulness of MRI in identifying remission of active CF [10, 11, 12].

The aim of this retrospective study was to evaluate the use of follow‐up MRI during CF management in terms of TCC treatment duration, time to full ambulation, and risk of future surgery.

## 2 Methods

All adult patients with diabetes mellitus diagnosed with active CF (ICD‐10 codes M90.8H, M90.8, M14.2, M14.6) between 2006 and 2022 at Skåne University Hospital (Lund and Malmö, Sweden) were considered for inclusion. Data were retrieved from medical records. Those who had inactive CF or a different diagnosis, as assessed by the treating physician, who did not have diabetes mellitus, who was not primarily treated with a non‐removable TCC, who did not have a diagnostic MRI, who died during CF treatment, or who did not give consent to study participation were thereafter excluded. The date when active CF was suspected and managed at our department was registered as the first visit. Age, sex, diabetes type and duration, HbA1c level, estimated glomerular filtration rate (eGFR), and history of peripheral arterial disease (PAD; defined as either previous lower limb revascularization or ankle‐brachial pressure index > 1.3 or < 0.9 in either leg) were recorded. eGFR was calculated according to the revised Lund‐Malmö formula based on creatinine level [13]. Renal impairment was defined as eGFR < 60 mL/min/1.73 m^2^. Biochemistry analyzed as close as possible to the first visit, not exceeding 1 year, was registered. Radiology reports from conventional X‐ray and MRI examinations were used to classify CF as stage 0 or 1 in accordance with Chantelau and Grützner [14]. Thus, cases of normal X‐ray and MRI with bone marrow edema (BMO) and without macrofractures or joint dislocation were classified as stage 0, and cases with the presence of macrofractures, joint dislocation, or skeletal deformity were classified as stage 1. Anatomical engagement was classified according to Sanders‐Frykberg [15].

Throughout the study period, management of active CF involved initial offloading with TCC until signs of inflammation diminished, a clinical decision which was supported by confirming a local temperature difference < 2°C compared with the corresponding area on the contralateral foot on two sequential visits. After ending TCC treatment, the general routine during the study period was to first transition the foot into a custom‐made orthotic walker, followed by therapeutic boot and finally therapeutic shoe at subsequent visits, at the discretion of the treating physician. Follow‐up X‐ray and MRI examinations were performed at the discretion of the physician, as there was no established routine or guideline detailing the use of follow‐up radiology examinations or assisting the interpretation of CF‐related MRI findings. Relapse of active CF was determined according to the assessment of the treating physician and generally suspected when temperature difference between feet was ≥ 2°C or radiology exams displayed worsened extent of CF‐related abnormalites.

Clinical follow‐up data, temperature measurements, X‐ray and MRI examinations, offloading modalities (TCC, orthotic walker, boot, and shoe), recurrences of active CF, and future ipsilateral surgery until December 31, 2023 were recorded. Findings from MRI examinations were compared to the proceeded examination using the radiologist's assessments. The condition was classified as worsened in the presence of more extensive BMO, new BMO, new dislocation, or new fracture. In the absence of any of these, the extension of BMO was compared to determine resolution, improvement, or no change. Comparisons between MRI examination outcomes, thermometry, and clinical decisions included visits with reliable clinical and thermometry records taking place within 45 days of the MRI examination and excluded cases of concurrent osteomyelitis. Results from three periods (2006–2012, 2013–2017, and 2018–2022) were analyzed and compared.

Categorical data are given as percentages. Continuous data are given as mean (SD) if normally distributed and otherwise as medians and interquartile ranges (IQRs) unless otherwise noted. Shapiro–Wilk test was used to test for normality. Categorical data were assessed using Fisher's exact or Chi‐squared test. Continuous data were assessed using independent samples *t*‐test or one‐way ANOVA if normally distributed, otherwise using the Mann–Whitney *U* test or Kruskal–Wallis test. A log‐rank test was used for assessing differences in time‐to‐event data. A multiple regression model was applied to evaluate factors that predicted time spent in TCC and time to full ambulation. A cut‐off of minimum 10 observations per predictor variable was used to prevent over‐fitting. Regression analyses with the outcome variable log‐transformed was performed if statistical assumptions for multiple regression were not met. Missing data were excluded list‐wise in the regression analyses. A *p*‐value < 0.05 was considered statistically significant. SPSS software (version 28; IBM Corporation, Chicago, IL) was used for statistical analyses.

This study was approved by the Swedish Ethical Review Authority (Dnr 2022‐05609‐01; Uppsala, Sweden).

### 2.1 Data and Resource Availability

Data analyzed in the current study are available from the corresponding author upon reasonable request.

## 3 Results

**FIGURE 1:**
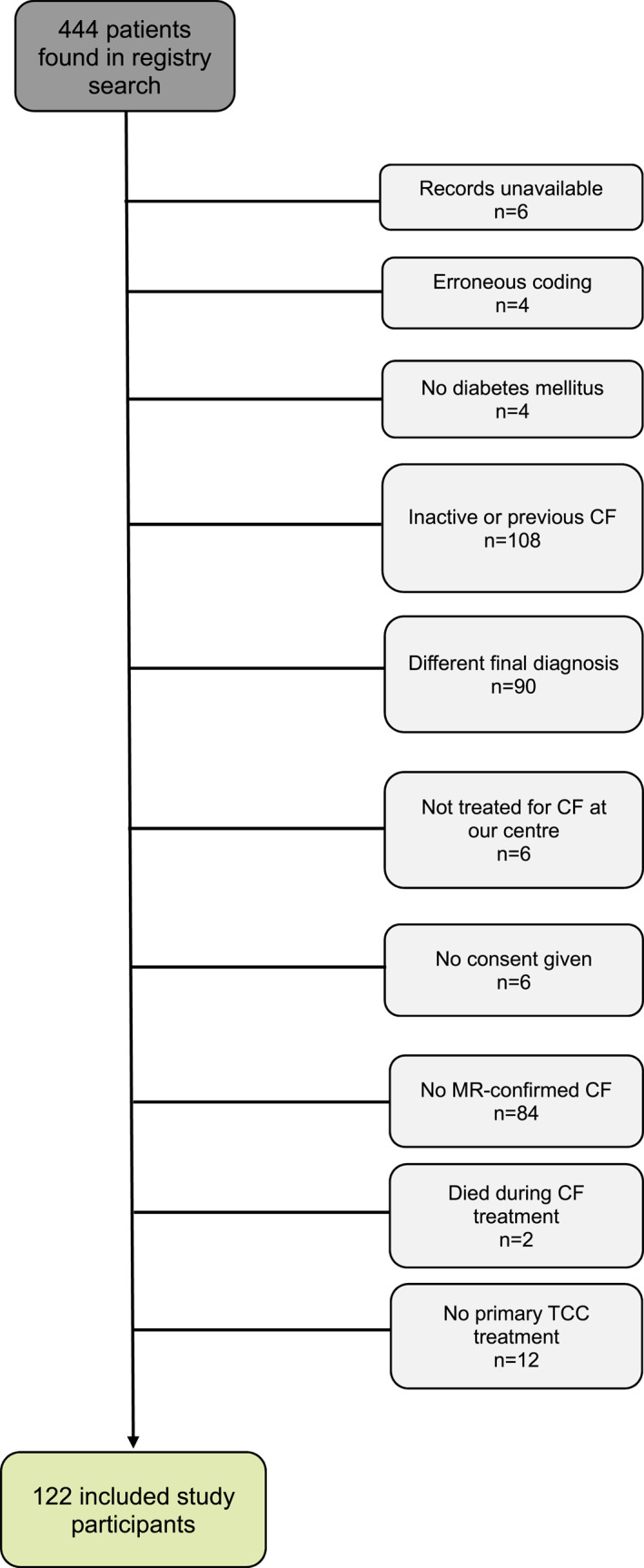
Flow chart of the inclusion of individuals in the study.

As shown in the study flow chart (Figure 1), 122 individuals with 143 CF events were included in this study. 56 individuals (47%) had an ankle brachial pressure index performed. Patient characteristics are given in Table 1. Of the 143 CF events, 67 (47%) were monitored clinically, with additional use of follow‐up X‐ray examinations in 34 events (51%). 76 events (53%) were monitored with MRI, with a total of 141 follow‐up MRI examinations (range, 1–7). The median time to the first follow‐up MRI examination from the first visit was 172 (110.5–274) days, and 16 (21%) of these first follow‐up examinations were ordered due to suspected relapse of active CF. More than one follow‐up MRI was conducted in 32 CF events (42% of CF events with follow‐up MRI examinations). The primary indications for using follow‐up MRI were suspected relapse of active CF in 39 cases (28%), suspected osteomyelitis in two cases (1.4%), and for monitoring purposes and aiding clinical follow‐up assessments in the remaining 100 cases (71%). The median number of days from follow‐up MRI to clinical assessment was 9 (5–20).

**TABLE 1:** Descriptive characteristics and outcomes of individuals and Charcot feet, in total and divided according to use of follow‐up MRI examinations.

	No follow‐up MRI	Follow‐up MRI	All	*p*
*n* (individuals)	56 (46%)	66 (54%)	122	
*n* (CF events)	67 (47%)	76 (53%)	143	
Right foot	33 (49%)	39 (51%)	72 (50%)	0.87
Female sex	16 (29%)	31 (47%)	47 (39%)	0.04
Age (years)	58 (13)	61 (14)	60 (13)	0.19
Type 1 DM	23 (41%)	22 (33%)	45 (37%)	0.45
Diabetes duration (years)	18 (11–31)	17 (8.0–30)	17 (10–30)	0.55
eGFR (60 mL/min/1.73 m^2^)^a^	67 (27)	68 (27)	68 (27)	0.84
eGFR below 60 mL/min/1.73 m^2^ ^a^	20 (36%)	27 (42%)	47 (39%)	0.57
HbA1c (mmol/mol)^b^	64 (52–77.5)	61 (51–75)	62 (52–75)	0.54
HbA1c (%)	8.0 (6.9–9.2)	7.7 (6.8–9.0)	7.8 (6.9–9.0)	0.54
HbA1c above 70 mmol/mol (8.6%)^b^	20 (36%)	20 (32%)	40 (34%)	0.70
PAD	11 (20%)	14 (21%)	25 (21%)	1.00
Sanders‐Frykberg 1^c^	6 (9.0%)	7 (9.2%)	13 (9.1%)	1.00
Sanders‐Frykberg 2^c^	38 (57%)	44 (58%)	82 (57%)	1.00
Sanders‐Frykberg 3^c^	41 (61%)	57 (75%)	98 (69%)	0.10
Sanders‐Frykberg 4^c^	6 (9.0%)	7 (9.2%)	13 (9.1%)	1.00
Sanders‐Frykberg 5^c^	6 (9.0%)	7 (9.2%)	13 (9.1%)	1.00
Stage 1 CF	39 (58%)	42 (55%)	81 (57%)	0.74
Concurrent ulceration at presentation	7 (10%)	8 (11%)	15 (10%)	1.00
Initial TCC duration (days)	72 (55–113)	137.5 (80–180.5)	101 (62–155)	< 0.001
Total TCC duration including recasting (days)	83 (56–133)	155 (89–235.5)	113 (64–195)	< 0.001
Feet re‐casted	12 (18%)	23 (30%)	35 (24%)	0.12
Feet re‐casted more than once	0	6 (7.9%)	6 (4.2%)	0.03
Time to full ambulation (days)^d^	276 (135.5–423)	445.5 (314–697)	367 (237–601)	< 0.001
Reconstructive surgery	2 (3.0%)	2 (2.6%)	4 (2.8%)	0.97
Partial amputation	9 (13%)	3 (3.9%)	12 (8.4%)	0.09
Toe amputation	5 (7.5%)	3 (3.9%)	8 (5.6%)	0.49
Metatarsal amputation	4 (6.0%)	0	4 (2.8%)	0.06
Above‐ankle amputation	5 (7.5%)	3 (3.9%)	8 (5.6%)	0.54
Lost to follow‐up^e^	2 (3.0%)	2 (2.6%)	4 (2.8%)	1.00
Follow‐up time (years)	6.8 (4.1–10)	4.7 (3.0–9.2)	5.7 (3.6–9.7)	0.01

*Note:* Continuous data given as median (IQR) or as mean (SD) if normally distributed.

^a^ Two cases with missing data in the follow‐up MRI group.

^b^ Three cases with missing data in the follow‐up MRI group.

^c^Some individuals presented with more than one anatomical location.

^d^ Two cases with missing data in the no follow‐up MRI group and 6 cases with missing data in the follow‐up MRI group.

^e^ In all cases, individuals were lost to follow‐up after end of TCC treatment.

In the comparison of skin temperature to the previous follow‐up MRI, nine visits were excluded (osteomyelitis in two cases, lack of thermometry in three cases, delay in four cases). In the remaining 132 visits, there was a discrepancy between MRI dynamics and infrared thermometry in 39 instances (30%; Tables S1–S3). In these cases, 17 clinical decisions (44%) were based on MRI dynamics when results were discrepant. This was especially common when the MRI result indicated a worsened condition despite a non‐significant temperature after transitioning to an orthotic walker.

**FIGURE 2:**
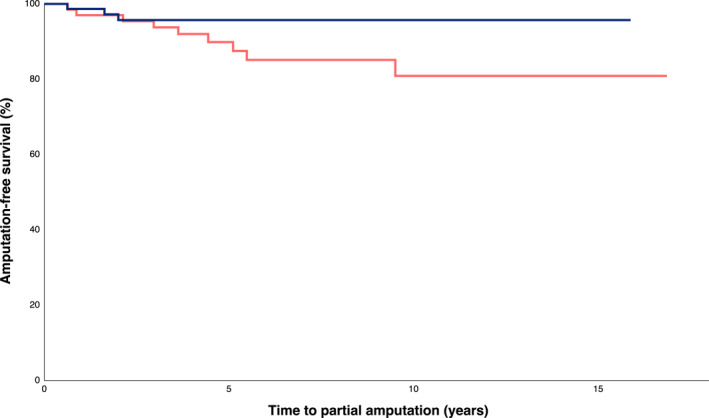
Risk of partial amputation after initiated CF treatment, divided according to the use of follow‐up MRI examinations (blue, follow‐up MRI group; red, no follow‐up MRI group).

Individuals monitored with follow‐up MRI had significantly longer initial TCC treatment durations (137.5 [80–180.5] vs. 72 [55–113] days, *p* < 0.001) and total TCC treatment durations including recasts (155 [89–235.5] vs. 83 [56–133] days, *p* < 0.001). The time to attain full ambulation in definitive shoewear was also significantly longer in these individuals (445.5 [314–697] vs. 276 [135.5–423] days, *p* < 0.001). A higher proportion of individuals in the Follow‐up MRI group were women (47% vs. 29%, *p* = 0.04), but other clinical characteristics, the prevalence of stage 1 CF at initiated offloading, and anatomical CF distributions were similar and did not significantly differ compared to those not monitored with follow‐up MRI. Individuals in the Follow‐up MRI group returned to TCC more often (30% vs. 18%, *p* = 0.12) and did so more than once in six cases, compared to none in the group not monitored with follow‐up MRI (*p* < 0.05). The rate of reconstructive surgery was similar between groups. The rates of partial and above‐ankle amputations were low, but numerically higher in the group not monitored with follow‐up MRI (9 [13%] vs. 3 [3.9%], *p* = 0.09; and 5 [7.5%] vs. 3 [3.9%], *p* = 0.54, respectively; Figure 2), but the follow‐up time was longer in this group (6.8 [4.1–10] vs. 4.7 [3.0–9.2] years, *p* < 0.05). The indication for partial amputation was infection in all three cases of the Follow‐up MRI group; in the No follow‐up MRI group, indications include infection (seven cases [78%]), ischemia (one case [11%]) and toe deformity (one case [11%]). Infection was the indication for every above‐ankle amputation in both groups.

Table 2 compares patient characteristics, assessments, and CF outcomes over the three time periods. The prevalence of stage 1 CF was similar in all three time periods, but the use of follow‐up MRI and TCC treatment duration increased from the first to the third period, and the use of follow‐up X‐ray examinations decreased. Over time, the proportion of individuals with type 1 diabetes mellitus decreased, as did the proportion of patients with an HbA1c > 70 mmol/mol.

**TABLE 2:** Descriptive characteristics and outcomes of individuals and Charcot feet, divided into chronological periods based on the date of the first visit.

	2006–2012	2013–2017	2018–2022	*p*
*n* (individuals)	31	41	50	
*n* (CF events)	35	48	60	
Age	56 (12) years	60 (13) years	61 (13) years	0.34
Female sex	14 (45%)	18 (44%)	15 (30%)	0.27
HbA1c (mmol/mol)	70 (62–80)	59 (52–79)	59 (49–70)	0.04
HbA1c (%)	8.6 (7.8–9.5)	7.5 (6.9–9.4)	7.5 (6.6–8.6)	0.04
HbA1c over 70 mmol/mol (8.6%)	14 (45%)	14 (35%)	12 (25%)	0.18
eGFR < 60 mL/min/1.73 m^2^	12 (39%)	16 (39%)	19 (40%)	1.00
Type 1 DM	17 (55%)	12 (29%)	16 (31%)	0.05
Diabetes duration	20 (13–39)	18 (8.5–25)	16 (8–27)	0.10
PAD	5 (16%)	11 (27%)	9 (18%)	0.46
Follow‐up x‐ray	26 (74%)	19 (40%)	18 (30%)	< 0.001
Follow‐up MRI	12 (34%)	24 (50%)	40 (67%)	0.01
Stage 1 CF	19 (54%)	27 (56%)	35 (58%)	0.93
Initial TCC duration (days)	69 (56–132)	115.5 (70–175)	104 (61–161.5)	0.04
Total TCC duration including recasting (days)	72 (61–135)	139.5 (70–217)	131.5 (71–197)	0.045
Feet re‐casted	6 (17%)	13 (27%)	16 (27%)	0.51

*Note:* Continuous data given as median (IQR) or as mean (SD) if normally distributed.

In a multiple regression model using logarithm‐adjusted dependent variables to achieve a normal distribution of residuals (Tables S4–S6) and including the use of follow‐up MRI, CF stage, time period, HbA1c > 70 mmol/mol, eGFR < 60 mL/min/1.73 m^2^, PAD, age, diabetes type, diabetes duration, and sex, only the use of follow‐up MRI (*p* < 0.001) was a statistically significant predictor of longer primary and total TCC treatment durations. Use of follow‐up MRI (*p* < 0.001) and CF stage 1 (*p* < 0.05) were statistically significant predictors of longer time to full ambulation. Sanders‐Frykberg anatomical localizations were not included in these analyses, as the number of observations per predictor variable would have been below 10. However, in a separate regression model that included only Sanders‐Frykberg localizations, no localization contributed significantly to the model (Tables S7–S9).

## 4 Discussion

In this retrospective cohort study, individuals monitored with follow‐up MRI examinations spent significantly longer time offloaded in TCC and had longer time to full ambulation compared to those not monitored with follow‐up MRI, despite similar individual characteristics and outcomes in terms of the need for surgical interventions. In regression analyses, only the use of follow‐up MRI and CF stage 1 independently predicted longer treatment times. Diagnosing CF in a later phase is well recognized to be associated with longer offloading and worse outcomes compared to an earlier diagnosis [2, 3, 4, 5]. However, the usefulness of follow‐up MRI in determining remission of active CF is based on more uncertain grounds. International guidelines from 2023 suggest that clinical findings, skin temperature measurements, and imaging should be used when assessing whether active CF is in remission, and also concluded that a paucity of studies and a lack of an established gold standard for defining remission made recommendations more difficult to establish [7].

A few previous studies have illustrated the use of follow‐up MRI in CF patients [10, 11, 12]. Zampa et al. [10] prospectively evaluated both the dynamics of BMO signal intensity and contrast enhancement at 3‐month intervals in 40 individuals with CF who were either receiving TCC or using orthotic walkers. The contrast uptake rate (CUR) decreased significantly and correlated well with temperature reduction after 3 months in 90% of cases, but most of these cases displayed retention or an increase in BMO signal intensity. The BMO signal intensity exhibited higher observer variability than CUR. The initial CUR was proportional to healing time, and clinical signs of resolution preceded MRI findings in 22.5% of cases. In a study by Chantelau [11], 45 cases of acute CF in 37 individuals monitored with follow‐up MRI were reviewed retrospectively with no established protocol of interval of its use. A decreased BMO signal intensity was observed in 69% of cases during follow‐up, and in most cases TCC treatment was stopped before complete resolution of BMO. The authors suggest using complementary X‐ray or CT examinations in each individual case to differentiate whether BMO reflects a destructive or reparative process. Additionally, a small study by Schlossbauer et al. reported strong correlations between BMO intensity and contrast enhancement in 13 individuals [12]. Finally, in a randomized feasibility study exploring outcomes, eligibility, and acceptability of serial MRI with 3‐month intervals in 30 individuals with confirmed CF, the duration of offloading was numerically longer (297 vs. 235 days, *p* = 0.096) for those randomized to serial MRI follow‐up.

There are several plausible explanations for the prolonged offloading with TCC in the MRI follow‐up group in this study. First, differences in clinical characteristics between groups may have been beyond what is reflected in the gathered data. For example, physicians might have been more willing to order follow‐up MRI for individuals assessed to have a more difficult CF episode or worse compliance. However, the rate of stage 1 CF, that is, the rate of individuals presenting with macrofractures and dislocations, did not differ between the groups. Second, clinicians might have awaited MRI results before deciding about offloading, and MRI examinations might have been scheduled later than requested in some cases. Third, discrepancies between temperature measurements and MRI dynamics were common and might have contributed to prolonged offloading if clinicians were reluctant to ease offloading until both parameters showed improvement despite improved clinical findings. The lack of consensus and established practice guidelines for using follow‐up MRI and interpreting BMO dynamics in CF could also have contributed to prolonged offloading treatment. Today, there is a well‐described retention of BMO in some cases of CF deemed to be in clinical resolution (9, 10), a phenomenon that clinicians might not have been fully aware of, especially early in the study period, which could have led to hesitancy against promoting reduced offloading despite clinical signs of resolution.

The rates of surgical interventions were low and no statistically significant differences between groups were seen. The group not monitored with follow‐up MRI had a numerically higher rate of partial amputations (*p* = 0.09; *p* = 0.06 for metatarsal amputations). If this result mirrors a lack of statistical power, possible explanations include longer follow‐up time and shorter offloading periods in the No follow‐up MRI group, which in combination could have contributed to more cases with ulcerations and infections during the study period.

If the use of follow‐up MRI in the treatment of CF indeed leads to lengthened offloading and more recasts, without obvious benefit otherwise, it would be contrary to the aim of using MRI to follow the course of disease better and avoid unnecessary offloading. In this study, only a minority of examinations were requested due to suspected relapse of active CF or osteomyelitis, the remainder were performed as serial examinations to aid in decision‐making at future clinical follow‐up visits. The overall similarity between the individuals in the follow‐up MRI and no follow‐up MRI groups, as well as the trend of longer offloading duration with increased use of follow‐up MRI between the studied periods, suggest that using follow‐up MRI in CF, as implemented in this study, inherently contributed to longer offloading with TCC. It can be argued that these findings indicate that follow‐up MRI primarily should be ordered in cases when either relapse of active CF or a concomitant diagnosis (e.g., infection) is suspected clinically, as a more differential diagnostic use of MRI seems more validated and easier to interpret [6, 16].

The strength of this study is its large cohort size, long follow‐up time, and minimal loss to follow‐up. This study also has several weaknesses. Its design is retrospective and observational, and no conclusion about causality regarding the use of follow‐up MRI can be made. Furthermore, the retrospective design could introduce bias, and data gathering depended on available documentation in medical records. The presence or development of ulceration and its relation to CF deformity and TCC treatment were not consistently available in descriptions, and ulceration as an outcome was therefore not included in this study. The study is based on individuals enrolled at a single center, perhaps reducing generalizability to different countries and healthcare systems. The dynamics of BMO between MRI examinations were broadly categorized, as the findings reported by radiologists were described in such wording, and the BMO signal intensity was typically assessed subjectively; it is possible that a more standardized and objective measurement approach by a radiologist could have contributed with more information. However, the study's design aimed to reflect the real‐world practice of clinicians using the radiologist's descriptions in MRI reports. Finally, the contrast enhancement on MRI was not consistently used or described in radiology reports and could, therefore, not be included as a parameter in this study.

In conclusion, in this retrospective cohort real‐world CF study, the Follow‐up MRI group displayed longer TCC and total CF treatment duration as compared to the No MRI follow‐up group despite relatively similar patient and foot characteristics and outcomes. Prospective, preferably randomized studies are needed to further elucidate the optimal use of MRI in monitoring CF, and contrast‐enhanced MRI should be studied in addition to BMO dynamics. While follow‐up MRI examinations should be considered when concurrent infection or relapse of active CF is suspected, this study suggests that caution should be taken when considering serial MRI examinations during CF treatment.

## Author Contributions

**Johan Schoug:** conceptualization, data curation, formal analysis, investigation, methodology, writing – original draft, writing – review and editing. **Per Katzman:** writing – review and editing. **Erik Uddman:** writing – review and editing. **Magnus Löndahl:** methodology, project administration, funding acquisition, supervision, writing – review and editing.

## Ethics Statement

This study was approved by the Swedish Ethical Review Authority (Dnr 2022‐05609‐01; Uppsala, Sweden).

## Conflicts of Interest

Until April 2024, M.L. has been on the speakers’ list and has received consultant fees from Abbott, Amgen Inc, AstraZeneca, Bayer, Boehringer‐Ingelheim, Eli Lilly and Company, Merck, Novartis, Novo Nordisk and Sanofi. M.L. has received unrestricted research grants from Boehringer‐Ingelheim and Sanofi. From May 2024, M.L. is an employee of Eli Lilly and Company. J.S. has nothing to disclose. P.K. has nothing to disclose. E.U. is on the speakers’ list for Eli Lilly and Company.

## Supporting information

Tables S1–S3

Tables S4–S9

## Data Availability

Data analyzed in the current study are available from the corresponding author upon reasonable request.
