# Supplementary material for: Follow‐Up Magnetic Resonance Imaging in Monitoring Charcot Foot and Its Association With Total Contact Cast Treatment Duration and Long‐Term Outcomes: A Retrospective Cohort Study
Source: J Foot Ankle Res. 2025 Jun 21;18(2):e70058. doi: 10.1002/jfa2.70058 (PMC12182253; doi:10.1002/jfa2.70058)
Supplement: Supplementary file 1 — Tables S1–S3 [file JFA2-18-e70058-s002.docx]

|  | Vanished BMO | | Reduced BMO | | Persisting BMO | | Worsened/new BMO/new fracture/new dislocation | |
| --- | --- | --- | --- | --- | --- | --- | --- | --- |
|  | < 2° C | ≥ 2° C | < 2° C | ≥ 2° C | < 2° C | ≥ 2° C | < 2° C | ≥ 2° C |
| n (exams) | 4 | 4 | 8 | 11 | 2 | 4 | 3 | 12 |
| n (CF events) | 4 | 4 | 8 | 11 | 2 | 4 | 3 | 8 |
| - Stage 1 CF | 1 (25%) | 3 (75%) | 3 (38%) | 9 (82%) | 1 (50%) | 3 (75%) | 1 (33%) | 3 (37%) |
| Time since first visit (days) | 213.5 (101.5-490; NA; 78-568) | 243 (136-561; NA; 131-636) | 115.5 (85-144; NA; 71-150) | 145 (97-209; 72-578; 69-647) | 197 (min/max 85-309) | 174.5 (121-597; NA; 119-722) | 197 (min/max 177-201) | 205 (115-399; 52-825; 52-875) |
| Time in TCC, current episode (days) | 125 (40.5-258; NA; 28-258) | 141 (59-288; NA; 35-334) | 107.5 (85-144; NA; 71-150) | 145 (85-209; 72-285; 69-300) | 182 (min/max 71-293) | 129.5 (121-200.5; NA; 119-223) | 177 (min/max 29-201) | 108 (49-199; 19-214; 18-217) |
| Time in TCC, cumulative (days) | 129.5 (80-385; NA; 78-456) | 201.5 (136-313.5; NA; 131-334) | 107.5 (85-144; NA; 71-150) | 145 (85-209; 72-506; 69-557) | 182 (71-293) | 174.5 (121-261; NA; 119-274) | 177 (169-201) | 194 (81-241; 26-308; 18-311) |
| MRI due to suspected reactivation | 1 (25%) | 1 (25%) | 0 | 0 | 1 (50%) | 0 | 1 (33%) | 2 (17%) |
| Recast within 90 days | 0 | 0 | 0 | 1 (10%) | 0 | 0 | 1 (33%) | 0 |
| Recast within 180 days | 0 | 0 | 0 | 1 (10%) | 0 | 0 | 1 (33%) | 2 (17%) |
| Recast within 1 year | 0 | 0 | 1 (12.5%) | 1 (10%) | 0 | 1 (25%) | 1 (33%) | 5 (42%) |
| Future days in TCC | 11.5 (0-42.5;NA;0-49) | 45.5 (21-126; NA; 21-145) | 0 (0-34.5; NA; 0-42) | 56 (28-98; 3-131; 0-133) | 14 (min/max 0-28) | 146 (50.5-230; NA; 28-249) | 47 (min/max 0-180) | 131 (68-282; 36-532; 28-557) |
| Time until full ambulation in footwear | 89.5 (12-231; NA; 0-265) | 180 (77-523; NA; 70-610) | 318.5 (318.5; 157-765; 0-920) | 733.5 (250-986; 92-1080; 77-1088) | 514 (min/max 154-874) | 1060 (684-1300; NA; 571-1367) | 329 (min/max 254-404) (one missing case) | 1118 (388-1698; NA; 78-2219) (four missing cases) |
| Continued TCC | 0 | 2 (50%) | 2 (25%) | 8 (73%) | 0 | 3 (75%) | 1 | 12 (100%) |
| - Future days in TCC | --- | 107.5 (70-145) | 38.5 (min/max 35-42) | 80.5 (35-117; NA; 28-133) | --- | 174 (min/max 118-249) | 56 | 131 (68-282; 36-532; 28-557) |
| - Time until full ambulation in footwear (days) | --- | 340 (70-610) | 491 (min/max 125-857) | 791.5 (293-1001; NA; 77-1088) | --- | 1023 (min/max 571-1098) | 404 | 1118 (388-1698; NA; 78-2219) (four missing cases) |
| Discontinued TCC | 4 (100%) | 2 (50%) | 6 (75%) | 3 (27%) | 2 (100%) | 1 (25%) | 2 (67%) | - |
| - Recast within 90 days | 0 | 0 | 0 | 1 (33%) | 0 | 0 | 0 |  |
| - Recast within 180 days | 0 | 0 | 0 | 1 (33%) | 0 | 0 | 0 |  |
| - Recast within 1 year | 0 | 0 | 1 (17%) | 1 (33%) | 0 | 0 | 0 |  |
| - Future days in TCC | 11.5 (0-42.5; NA; 0-49) | 21 (21-21) | 0 (0-8; NA; 0-33) | 13 (NA; NA; 0-33) | 14 (min/max 0-28) | 28 | 90 (0-180) |  |
| - Time until full ambulation in footwear | 89.5 (12-231; NA; 0-265) | 180 (98-262) | 318.5 (189-597.5; NA; 0-920) | 351 (NA; NA; 230-804) | 514 (min/max 154-874) | 1367 | 254 (one missing case) |  |

Supplemental Table 1. Comparison of skin temperature readings with preceding follow-up MRI result in CF offloaded with total contact cast (TCC). Data are given as median (25-75^th^ percentile, 10-90^th^ percentile, minimum-maximum unless otherwise noted.

|  | Vanished BMO | | Reduced BMO | | Persisting BMO | | Worsened/new BMO/new fracture/new dislocation | |
| --- | --- | --- | --- | --- | --- | --- | --- | --- |
|  | < 2° C | ≥ 2° C | < 2° C | ≥ 2° C | < 2° C | ≥ 2° C | < 2° C | ≥ 2° C |
| n (exams) | 11 | 6 | 15 | 3 | 5 | 6 | 7 | 8 |
| n (CF events) | 11 | 6 | 15 | 3 | 5 | 4 | 7 | 8 |
| - Stage 1 CF | 6 (55%) | 3 (50%) | 12 (80%) | 2 (67%) | 3 (60%) | 2 (50%) | 5 (71%) | 5 (62.5%) |
| Time since first visit (days) | 447 (210-861; 119-1185; 98-1193) | 393 (220-717; NA; 167-1104) | 334 (214-427; 154-726; 147-900) | 498 (133-913 min/max) | 601 (464.5-1283; NA; 411-1491) | 404 (284-731; NA; 221-822) | 235 (185-633; NA; 185-1644) | 376.5 (258.5-570; NA; 183-973) |
| Time in TCC, cumulative (days) | 166 (92-356; 32-519; 22-545) | 205.5 (97.5-298, NA, 54-364) | 148 (100-171; 67-212; 66-220) | 239 (62-304) | 315 (217.5-480.5; NA; 172-639) | 124 (101-195; NA; 96-278) | 175 (86-335; NA; 31-526) | 155 (137-312; NA; 109-381) |
| Time since uncasting (days) | 147 (65-398; 35-1062; 34-1171) | 178 (54-458; NA; 29-867) | 176 (64-334; 36-380; 28-435) | 163 (min/max 81-261) | 238 (202.5-532; NA; 174-741) | 288 (109-444.5; NA; 38-539) | 142 (34-485; NA; 16-693) | 103.5 (57-325; NA; 42-448) |
| MRI due to suspected reactivation | 3 (27%) | 2 (33%) | 5 (33%) | 1 (33%) | 2 (40%) | 3 (50%) | 4 (57%) | 5 (62.5%) |
| Recast within 90 days | 0 | 0 | 0 | 0 | 0 | 1 (17%) | 0 | 0 |
| Recast within 180 days | 1 (9.1%) | 0 | 0 | 0 | 0 | 1 (17%) | 2 (29%) | 0 |
| Recast within 1 year | 1 (9.1%) | 0 | 1 (6.7%) | 0 | 0 | 1 (17%) | 2 (29%) | 1 (12.5%) |
| Future days in TCC | 0 (0-0; 0-153; 0-191) | 0 (0-0; 0-0; 0-0) | 0 (0-0; 0-70; 0-154) | 0 (0-0) | 0 (0-20.5; NA; 0-41) | 0 (0-47.5; NA; 0-190) | 0 (0-102; NA; 0-281) | 56.5 (0-227; NA; 0-332) |
| Time until full ambulation in footwear | 117.5 (62.5-559; 30-1392; 49-364) (one missing) | 145.5 (49-428; NA; 28-511) (two missing) | 162 (85-582.5;25-1424;0-1794) (two missing) | 719 (0-1176) | 235 (73.5-702; NA; 69-806) | 404 (NA; NA; 210-490) (3 missing) | 544 (105-713; NA; 98-966) (one missing) | 692 (380-1039.5; NA; 270-1500) (two missing) |
| Continued orthosis | 5 (45.5%) | 3 (50%) | 6 (40%) | 3 (100%) | 5 (100%) | 5 (83%) | 7 (100%) | 3 (37.5%) |
| - Recast within 90 days | 0 | 0 | 0 | 0 | 0 | 0 | 0 | 0 |
| - Recast within 180 days | 1 (20%) | 0 | 0 | 0 | 0 | 0 | 2 (29%) | 0 |
| - Recast within 1 year | 1 (20%) | 0 | 1 (17%) | 0 | 0 | 0 | 2 (29%) | 0 |
| - Future days in TCC | 0 (0-95.5; NA; 0-191) | 0 (0-0) | 0 (0-0) | 0 (0-0) | 0 (0-20.5; NA; 0-41) | 0 (0-0) | 0 (0-102; NA; 0-281) | 0 (0-0) |
| - Time until full ambulation in footwear (days) | 108.5 (63-1129; NA; 61-1457) (one missing) | 103 (NA; NA; 28-178) (one missing) | 100 (86-582.5; NA; 73-857) (one missing) | 719 (0-1176) | 235 (73.5-702; NA; 69-806) | 404 (NA; NA; 210-490) (3 missing) | 544 (105-713; NA; 98-966) (one missing) | 692 (NA; NA; 690-694) (one missing) |
| Restarted TCC | 0 | 0 | 0 | 0 | 0 | 1 (17%) | 0 | 5 (62.5%) |
| - Future days in TCC |  |  |  |  |  | 190 |  | 154 (56.5-291.5; NA; 55-332) |
| - Time until full ambulation in footwear |  |  |  |  |  | NA |  | 651.5 (307-1346.5; NA; 270-1500) (one missing) |
| Introduced footwear | 6 (54.5%) | 3 (50%) | 9 (60%) | 0 | 0 | 0 | 0 | 0 |
| - Recast within 90 days | 0 | 0 | 0 |  |  |  |  |  |
| - Recast within 180 days | 0 | 0 | 0 |  |  |  |  |  |
| - Recast within 1 year | 0 | 0 | 1 (11%) |  |  |  |  |  |
| - Future days in TCC | 0 (0-0) | 0 (0-0) | 0 (0-7;NA;0-154) |  |  |  |  |  |
| - Time until full ambulation in footwear | 222 (54-559;NA;27-812) | 312 (NA;NA;113-511) (one missing) | 187 (71.5-727;NA;0-1794) (one missing) |  |  |  |  |  |

Supplemental Table 2. Comparison of skin temperature readings with preceding follow-up MRI result in CF equipped with an orthotic walker. Data are given as median (25-75^th^ percentile, 10-90^th^ percentile, minimum-maximum unless otherwise noted.

|  | Vanished BMO | | Reduced BMO | | Persisting BMO | | Worsened/new BMO/new fracture/new dislocation | |
| --- | --- | --- | --- | --- | --- | --- | --- | --- |
|  | < 2° C | ≥ 2° C | < 2° C | ≥ 2° C | < 2° C | ≥ 2° C | < 2° C | ≥ 2° C |
| n (exams) | 3 | 0 | 7 | 1 | 3 | 1 | 4 | 4 |
| n (CF cases) | 3 | 0 | 7 | 1 | 3 | 1 | 4 | 4 |
| - Stage 1 CF | 3 (100%) |  | 3 (43%) | 1 (100%) | 2 (67%) | 1 (100%) | 2 (50%) | 2 (50%) |
| Time since first visit | 450 (min/max 288-1013) |  | 308 (128-685;NA;70-903) | 503 | 160 (NA;NA;141-1225) | 336 | 376.5 (258.5-605.5;NA;249-652) | 305.5 (178-644;NA;165-727) |
| Time in TCC, cumulative | 120 (33-346 min/max) |  | 93 (72-327;NA;33-344) | 203 | 32 (NA;NA;22-306) | 241 | 136 (83-311;NA;80-355) | 126 (99.5-230.5;NA;98-258) |
| Time since uncasting | 433 (175-680 min/max) |  | 224 (70-352;NA;42-566) | 176 | 294 (NA;NA;113-475) | 12 | 245 (189-306;NA;187-310) | 182.5 (58-438;NA;50-490) |
| MRI due to suspected reactivation | 1 (33%) |  | 1 (14%) | 1 (100%) | 0 | 0 | 2 (50%) | 2 (50%) |
| Recast within 90 days | 0 |  | 0 | 0 | 0 | 1 (100%) | 0 | 0 |
| Recast within 180 days | 0 |  | 0 | 0 | 0 |  | 0 | 0 |
| Recast within 1 year | 0 |  | 0 | 0 | 0 |  | 0 | 0 |
| Future days in TCC | 0 (0-0) |  | 0 (0-0) | 192 | 0 (0-0) |  | 0 (0-0) | 65.5 (6.5-126;NA;0-133) |
| Time until full ambulation in footwear | 91 (0-98 min/max) |  | 171 (0-419;NA;0-696) | 636 | 482 (100-864) |  | 85 (12-485.5;NA;0-607) | 182.5 (58-438;NA;208-490) (one missing) |
| Restarted TCC | 0 | - | 0 | 0 | 0 | 1 (100%) | 0 | 3 (75%) |
| - Future days in TCC |  |  |  |  |  | 27 |  | 105 (26-133 min/max) |
| - Time until full ambulation in footwear |  |  |  |  |  | 98 |  | 213 (208-218 min/max) (one missing) |
| Restarted orthosis | 0 | - | 1 (14%) | 1 (100%) | 0 |  | 1 (25%) | 1 (25%) |
| - Recast within 90 days |  |  | 0 | 0 |  |  | 0 | 0 |
| - Recast within 180 days |  |  | 0 | 0 |  |  | 0 | 0 |
| - Recast within 1 year |  |  | 0 | 0 |  |  | 0 | 0 |
| - Future days in TCC |  |  |  | 192 |  |  | 0 | 0 |
| - Time until full ambulation in footwear |  |  |  | 636 |  |  | 607 | 490 |
| Continued footwear | 3 (100%) | - | 6 (86%) | 0 | 3 (100%) |  | 3 (75%) | 0 |
| - Recast within 90 days | 0 |  | 0 |  | 0 |  | 0 |  |
| - Recast within 180 days | 0 |  | 0 |  | 0 |  | 0 |  |
| - Recast within 1 year | 0 |  | 0 |  | 0 |  | 0 |  |
| - Future days in TCC | 0 (0-0) |  | 0 (0-0) |  | 0 (0-0) |  | 0 (0-0) |  |
| - Time until full ambulation in footwear | 91 (0-98 min/max) |  | 171 (0-419;NA;0-696) |  | 482 (100-864) |  | 49 (0-121 min/max) |  |

Supplemental Table 3. Comparison of skin temperature readings with preceding follow-up MRI result in CF equipped with boots or therapeutic shoes. Data are given as median (25-75^th^ percentile, 10-90^th^ percentile, minimum-maximum unless otherwise noted.
