# Supplementary material for: Follow‐Up Magnetic Resonance Imaging in Monitoring Charcot Foot and Its Association With Total Contact Cast Treatment Duration and Long‐Term Outcomes: A Retrospective Cohort Study
Source: J Foot Ankle Res. 2025 Jun 21;18(2):e70058. doi: 10.1002/jfa2.70058 (PMC12182253; doi:10.1002/jfa2.70058)
Supplement: Supplementary file 2 — Tables S4–S9 [file JFA2-18-e70058-s001.docx]

|  | Unstandardized beta coefficient | Standard error of beta coefficient | 95% CI for beta | *P* |
| --- | --- | --- | --- | --- |
| Constant | 1.73 | 0.18 | 1.38, 2.07 | < 0.001 |
| Chronological period | 0.013 | 0.04 | -0.06, 0.08 | 0.72 |
| Stage 1 CF | 0.11 | 0.06 | -0.003, 0.22 | 0.06 |
| Follow-up MRI use | 0.19 | 0.06 | 0.08, 0.30 | 0.001 |
| HbA1c > 70 mmol/mol | 0.02 | 0.06 | -0.09, 0.14 | 0.69 |
| PAD | -0.09 | 0.07 | -0.22, 0.04 | 0.17 |
| Age | -0.002 | 0.002 | -0.006, 0.003 | 0.51 |
| eGFR < 60 ml/min/1.73m^2^ | -0.08 | 0.06 | -0.19, 0.04 | 0.19 |
| Type 2 diabetes | 0.15 | 0.08 | -0.01, 0.31 | 0.07 |
| Diabetes duration | 0.005 | 0.003 | 0.001, 0.01 | 0.047 |
| Sex | 0.01 | 0.07 | -0.10, 0.13 | 0.84 |

Supplemental Table 4. Multiple regression using 10-logarithmed primary TCC time as dependent variable (n=119 individuals).

|  | Unstandardized beta coefficient | Standard error of beta coefficient | 95% CI for beta | *P* |
| --- | --- | --- | --- | --- |
| Constant | 1.79 | 0.19 | 1.41, 2.16 | < 0.001 |
| Chronological period | 0.02 | 0.04 | -0.06, 0.10 | 0.62 |
| Stage 1 CF | 0.11 | 0.06 | -0.01, 0.23 | 0.07 |
| Follow-up MRI use | 0.22 | 0.06 | 0.10, 0.34 | < 0.001 |
| HbA1c > 70 mmol/mol | 0.05 | 0.06 | -0.07, 0.18 | 0.40 |
| PAD | -0.11 | 0.07 | -0.25, 0.03 | 0.14 |
| Age | -0.004 | 0.003 | -0.009, 0.001 | 0.16 |
| eGFR < 60 ml/min/1.73m^2^ | -0.02 | 0.06 | -0.14, 0.11 | 0.80 |
| Type 2 diabetes | 0.16 | 0.09 | -0.02, 0.34 | 0.08 |
| Diabetes duration | 0.005 | 0.003 | -0.001, 0.01 | 0.09 |
| Sex | 0.05 | 0.06 | -0.08, 0.17 | 0.44 |

Supplemental Table 5. Multiple regression using 10-logarithmed total TCC time as dependent variable (n=119 individuals).

|  | Unstandardized beta coefficient | Standard error of beta coefficient | 95% CI for beta | *P* |
| --- | --- | --- | --- | --- |
| Constant | 2.64 | 0.18 | 2.27, 3.00 | < 0.001 |
| Chronological period | -0.03 | 0.04 | -0.11, 0.04 | 0.41 |
| Stage 1 CF | 0.13 | 0.06 | 0.01, 0.24 | 0.03 |
| Follow-up MRI use | 0.25 | 0.06 | 0.13, 0.37 | < 0.001 |
| HbA1c > 70 mmol/mol | -0.03 | 0.06 | -0.15, 0.09 | 0.65 |
| PAD | -0.08 | 0.07 | -0.22, 0.06 | 0.27 |
| Age | -0.005 | 0.002 | -0.01, 0.001 | 0.07 |
| eGFR < 60 ml/min/1.73m^2^ | 0.02 | 0.06 | -0.10, 0.14 | 0.70 |
| Type 2 diabetes | 0.08 | 0.09 | -0.09, 0.25 | 0.37 |
| Diabetes duration | 0.001 | 0.003 | -0.005, 0.005 | 0.99 |
| Sex | 0.008 | 0.06 | -0.11, 0.13 | 0.89 |

Supplemental Table 6. Multiple regression using 10-logarithmed time to full ambulation as dependent variable (n=113 individuals).

|  | Unstandardized beta coefficient | Standard error of beta coefficient | 95% CI for beta | *P* |
| --- | --- | --- | --- | --- |
| Constant | 1.98 | 0.07 | 1.83, 2.12 | <0.001 |
| Sanders Frykberg 1 | -0.10 | 0.10 | -0.29, 0.10 | 0.33 |
| Sanders Frykberg 2 | -0.07 | 0.06 | -0.18, 0.05 | 0.27 |
| Sanders Frykberg 3 | 0.08 | 0.06 | -0.04, 0.20 | 0.18 |
| Sanders Frykberg 4 | -0.11 | 0.10 | -0.30, 0.08 | 0.26 |
| Sanders Frykberg 5 | -0.003 | 0.09 | -0.19, 0.18 | 0.98 |

Supplemental Table 7. Multiple regression using 10-logarithmed primary TCC time as dependent variable (n = 143 CF events).

|  | Unstandardized beta coefficient | Standard error of beta coefficient | 95% CI for beta | *P* |
| --- | --- | --- | --- | --- |
| Constant | 2.00 | 0.08 | 1.85, 2.15 | < 0.001 |
| Sanders Frykberg 1 | -0.03 | 0.10 | -0.23, 0.18 | 0.81 |
| Sanders Frykberg 2 | -0.05 | 0.06 | -0.17, 0.07 | 0.40 |
| Sanders Frykberg 3 | 0.12 | 0.07 | -0.01, 0.24 | 0.07 |
| Sanders Frykberg 4 | -0.06 | 0.10 | -0.26, 0.14 | 0.55 |
| Sanders Frykberg 5 | -0.01 | 0.10 | -0.21, 0.18 | 0.88 |

Supplemental Table 8. Multiple regression using 10-logarithmed total TCC time as dependent variable (n = 143 CF events).

|  | Unstandardized beta coefficient | Standard error of beta coefficient | 95% CI for beta | *P* |
| --- | --- | --- | --- | --- |
| Constant | 2.56 | 0.08 | 2.40, 2.72 | < 0.001 |
| Sanders Frykberg 1 | -0.14 | 0.11 | -0.37, 0.08 | 0.21 |
| Sanders Frykberg 2 | -0.08 | 0.07 | -0.21, 0.06 | 0.25 |
| Sanders Frykberg 3 | 0.08 | 0.07 | -0.06, 0.21 | 0.27 |
| Sanders Frykberg 4 | 0.01 | 0.12 | -0.21, 0.24 | 0.91 |
| Sanders Frykberg 5 | -0.10 | 0.11 | -0.32, 0.12 | 0.36 |

Supplemental Table 9. Multiple regression using 10-logarithmed time to full ambulation as dependent variable (n = 135 CF events).
